# The effectiveness of low-dose desmopressin in improving hypothermia-induced impairment of primary haemostasis under influence of aspirin – a randomized controlled trial

**DOI:** 10.1186/s12871-015-0061-5

**Published:** 2015-05-28

**Authors:** Pui Yee Tsui, Chi Wai Cheung, Yvonne Lee, Susan Wai Sum Leung, Kwok Fu Jacobus Ng

**Affiliations:** 1 Department of Anaesthesia, Pamela Youde Nethersole Eastern Hospital, Chai Wan, Hong Kong, SAR China; 2 Department of Anaesthesiology, The University of Hong Kong, Room K424, 4th Floor, K Block, Queen Mary Hospital, 102 Pokfulam Road, Pokfulam, Hong Kong, SAR China; 3 Department of Pharmacology & Pharmacy, The University of Hong Kong, Hong Kong, SAR China

**Keywords:** Aspirin, Blood coagulation, Blood loss, Desmopressin, Hypothermia

## Abstract

**Background:**

Mild hypothermia (34–35 °C) increases perioperative blood loss. Our previous studies showed that desmopressin could have *in vitro* beneficial effects on hypothermia-induced primary haemostasis impairment. In this study, we investigate the *in vitro* effects of desmopressin on hypothermia-induced primary haemostasis impairment under the influence of aspirin in healthy volunteers.

**Methods:**

Sixty healthy volunteers were randomly allocated to taking aspirin 100 mg or placebo for three days. On the sixth day blood samples were taken before and after the injection of desmopressin (1.5 microgram or 5 microgram) or normal saline subcutaneously. Measurements including Platelet Function Analyzer (PFA-100®) closure times, plasma von Willebrand Factor antigen, haemoglobin and platelet levels were made at 32 °C and 37 °C respectively.

**Results:**

Collagen/epinephrine closure time (EPICT) was significantly prolonged by 21.13 % (95 %CI 2.34–39.74 %, *p* = 0.021) in aspirin group at 37 °C. While hypothermia alone prolonged both collagen/adenosine diphosphate (ADPCT) and EPICT by 17.63 % (95 %CI 13.5–20.85 %, *p* < 0.001) and 8.0 % (95 %CI 6.38–10.04 %, *p* = 0.024) respectively, addition of aspirin only further prolonged EPICT by 19.9 % (95 %CI 3.32–36.49 %, *p* = 0.013). In aspirin group, desmopressin 1.5 microgram and 5 microgram significantly reduced ADPCT to below baseline levels at 37 °C (*p* = 0.025 and <0.001 respectively), whereas reduction in EPICT was seen with desmopressin 5 microgram (*p* =0.008). The effect was less pronounced at 32 °C, with a significant reduction in EPICT obtained with a dosage of 5 microgram only (*p* = 0.011).

**Conclusion:**

It was shown that aspirin could further potentiate the hypothermia-induced closure time prolongations. Low dose desmopressin (1.5 microgram) reduced PFA-100® closure times towards baseline. A higher dosage (5 microgram) further reduced the closure times below baseline. Therefore low dose desmopressin (1.5 microgram) might have the potential to correct hypothermia-induced primary haemostasis impairment under the influence of aspirin during the perioperative period.

**Trial registration:**

ClinicalTrials.gov: NCT01382134

## Background

Aspirin is the most commonly taken anti-platelet drug. While it has roles in primary and secondary preventions in a variety of thromboembolic conditions, it can also lead to impairment in primary haemostasis. In particular, pre-operative aspirin intake has been associated with increased post-operative blood loss [1–3]. In the recent POISE-2 study, it was shown that peri-operative intake of aspirin (100-200 mg daily) increased risk of major bleeding in patients, while risk of death or non-fatal myocardial infarction was not reduced when compared to the placebo group [4]. There is always a dilemma on whether cessation of anti-platelet therapy during the peri-operative period would increase the risk of thromboembolic event, especially in patients with increased cardiovascular risks [5, 6]. Given the widespread use of aspirin in primary and secondary cardiovascular disease preventions, it is not uncommon that patients will be under its influence during the peri-operative period. On the other hand, intra-operative hypothermia may also have a negative impact on perioperative blood loss as well. It was previously shown that hypothermia (34–35 °C) increased surgical bleeding and transfusion requirements in both cardiac and non-cardiac surgery [7–10]. Hypothermia also plays a significant role in coagulopathy in trauma patients and those requiring massive transfusion [11, 12]. While hypothermia might be difficult to avoid totally, it was found that low dose desmopressin might have beneficial effect on the primary haemostasis impairment it causes [13, 14]. We therefore conducted this study to evaluate any effect of aspirin on hypothermia-induced primary haemostasis impairment, and whether low dose desmopressin could possibly influence such effect.

## Methods

The study was approved by the Hong Kong West Cluster/University of Hong Kong Institutional Review Board (Reference number: UW11-075) and registered at www.ClinicalTrials.gov with Trial Registration Number NCT01382134. The study was carried out according to the Declaration of Helsinki. Sixty healthy volunteers were recruited from August 2011 to June 2012 and informed consents were obtained from all of them. All subjects were over eighteen years old and had no known medical disease nor pregnant. Smokers, regular alcohol users and those with a known history of bleeding disorder were excluded. Subjects were instructed not to take any medication or herbal preparations that would affect haemostasis in the preceding two weeks. They were also instructed not to have red wine or chocolate intake 24 h before blood collection.

Subjects were randomized by computer into aspirin group, taking low dose aspirin 100 mg daily (Cartia 100 mg. Glaxo Smith Kline, Sigma Pharmaceuticals Pty Ltd, Dandenong, Australia) or placebo group, taking placebo daily for a total of three days. Fifteen males and fifteen females were assigned to the aspirin group, while thirteen males and seventeen females to the placebo group. On the fourth day, morning urine samples were collected for detection of urine 11-dehydro thromboxane B2 (11-dehydro thromboxane B2 EIA Kit Cat. no. 19501. Cayman Chemical Company, Michigan, USA) to check for response to aspirin. On the sixth day, subjects were invited to our laboratory for venous blood sampling and drug testing. After collection of baseline blood samples, subjects were randomized to receive subcutaneous injection of either 0.9 % sodium chloride (placebo), desmopressin 1.5 microgram or 5 microgram made up to 1 ml solution (Octostim 15microgram/ml Desmopressin Acetate. Ferring International Center SA, St Prex, Switzerland). Blood samples were collected two hours later from the contralateral arm for post-treatment analysis.

All blood sampling, drug administration and measurements were performed by investigators who were blinded to the randomization codes. The venous blood samples were stored in citrated samples (BD Vacutainer 0.109 M, 3.2 % Citrate Tube Cat. no. 363047. BD Diagnostics, Dubai, United Arab Emirates). The blood samples were then divided into two groups and subjected to *in-vitro* hypothermia (37 °C versus 32 °C) and haemodiution (undiluted versus dilution with 20 % by volume of normal saline). They were subjected to the following tests: (1) PFA-100® closure times (PFA-100 Platelet Function Analyzer. Dade Behring Inc, West Sacrameto, USA), using collagen/adenosine diphosphate (ADP) and collagen/epinephrine (EPI) cartridges as described previously [15] (Dade PFA collagen/EPI Test Cartridge Cat. no. B4170-20; Dade PFA collagen/ADP Test Cartridge Cat. no. B4170-21; Both from Siemens Healthcare Diagnostic Products GmbH, Marburg, Germany). They were incubated at 32 or 37 °C for 15 min before performing any PFA-100® measurements; (2) full blood count (pocH-100i Automated Hematology Analyzer. Sysmex Corporation, Kobe, Japan) and plasma fibrinogen (CA-50 Automated Blood Coagulation Analyzer. Sysmex Corporation, Kobe, Japan); (3) plasma von Willebrand Factor (vWF) antigen (Imubind vWF ELISA plate Cat. no. 828. American Diagnostica Inc, Stamford, US).

Repeated measures analysis of variance (RANOVA) and Chi-square tests were used in analysis of closure times within each group. Inter-group comparisons were analyzed using Mann–Whitney *U* test (for 2 independent groups) and Wilcoxon Signed-rank test (for 2 dependent groups) due to violation of normal distribution. Post-hoc comparisons were performed with paired *t*-test with Bonferroni correction. Statistical analysis was performed using SAS System for Windows Release 9.2, SAS Institute Inc., Cary, NC, USA. A *p* value of <0.05 is considered significant. Using a standard deviation of 16 % of the values of mean closure times obtained from other healthy volunteers at our centre, our study would have a power of 90 % for detection of a 25 % change in PFA-100® closure times with ten subjects in each group [16].

## Results

**Fig. 1:**
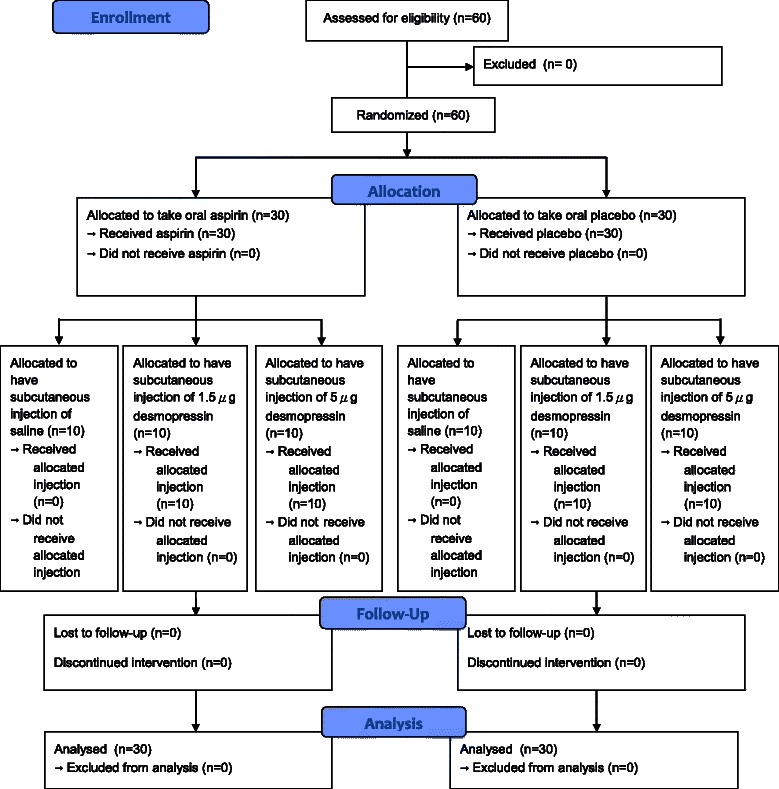
Flow diagram of patient recruitment

A total of sixty subjects were recruited in the study and all were included in data analysis (Fig. 1). One subject from the placebo group reported mild facial swelling and stomach discomfort later the day after having desmopressin injection, which was self-limiting and no serious adverse reactions were reported.

**Fig. 2:**
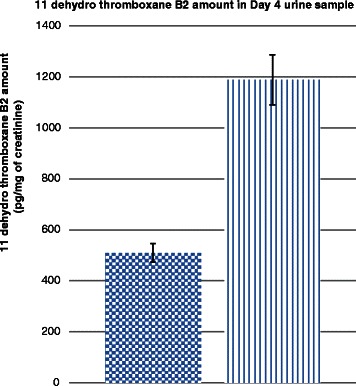
Levels of 11-dehydro thromboxane B2 in aspirin group and placebo group. () represents aspirin group and () represents placebo group. *p* < 0.001

Levels of urinary 11 dehydro thromboxane B2 in aspirin-taking subjects were significantly lower than that of placebo group (Fig. 2). Table 1 showed the baseline closure time measurements of both aspirin and placebo groups. The baseline EPICTs were significantly prolonged in aspirin group by 21.13 % (95 %Cl 2.34–39.74 %, *p* = 0.021) when compared with the placebo group. Hypothermia alone prolonged both ADPCT and EPICT by 17.63 % (95 %Cl 13.5–20.85 %, *p* < 0.001) and 8.0 % (95 %Cl 6.38–10.04 %, *p* = 0.024) respectively when compared to normothermic values. The combination of aspirin and hypothermia further prolonged EPICT by 19.9 % (95 %Cl 3.32–36.49 %, *p* = 0.013) when compared with hypothermia alone, but ADPCT was not significantly prolonged.

**Table 1:** Baseline closure times of aspirin and placebo groups at 37 °C

	Aspirin group 37 °C	Placebo group 37 °C	*p* value
	(*n* = 30)	(*n* = 30)	
ADPCT	120.7 ± 46.6	123.9 ± 42.5	0.728
EPICT	211.3 ± 68.9	174.6 ± 56.8	0.021^+^
	Aspirin group 32 °C	Placebo group 32 °C	*p* value
	(*n* = 30)	(*n* = 30)	
ADPCT	142.3 ± 58.2	145.7 ± 62.6	0.690
EPICT	226.1 ± 67.6	188.6 ± 52.5	0.013^+^

Values in mean ± SD

^+^Significant at the 0.05 level

ADPCT: Collagen/adenosine diphosphate closure time

EPICT: Collagen/adrenaline closure time

**Fig. 3:**
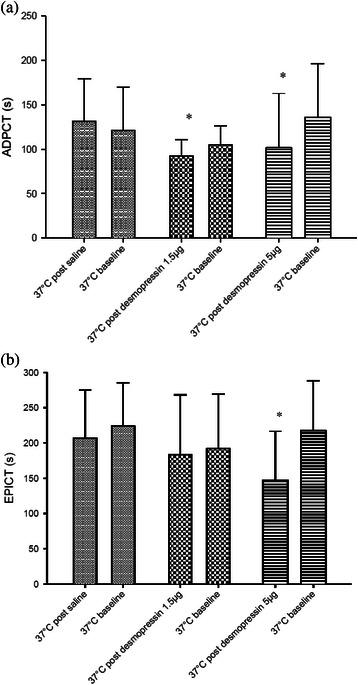
Effects of desmopressin on PFA-100® closure times ADPCT (**a**) and EPICT (**b**) of aspirin group at 37 °C. Bars represent control (normal saline) (), desmopressin 1.5 microgram () and 5 microgram () respectively. * indicates *p* < 0.05. Error bars indicate SD. ADPCT: Collagen/adenosine diphosphate closure time. EPICT: Collagen/adrenaline closure time

The administration of desmopressin significantly reduced both ADPCT and EPICT of aspirin group at 37 °C (Fig. 3). While there was significant reduction of ADPCT at doses 1.5 microgram (*p* = 0.025) and 5 microgram (*p* <0.001) when comparing to baseline values, an insignificant reduction of EPICT was seen with 1.5 microgram (*p* = 0.65) and significant reduction at 5 microgram (*p* = 0.008).

**Fig. 4:**
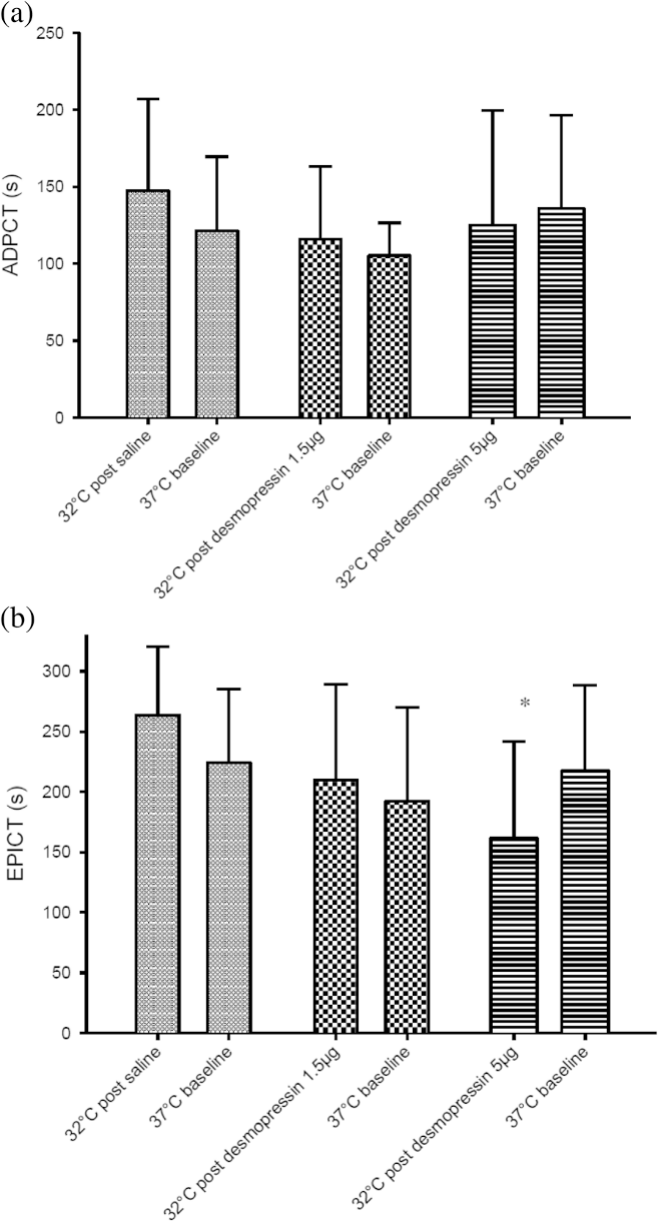
Effects of desmopressin on PFA-100® closure times ADPCT (**a**) and EPICT (**b**) of aspirin group at 32 °C. Bars represent control (normal saline) (), desmopressin 1.5 microgram () and 5 microgram () respectively. * indicates *p* < 0.05. Error bars indicate SD. ADPCT: Collagen/adenosine diphosphate closure time. EPICT: Collagen/adrenaline closure time

**Fig. 5:**
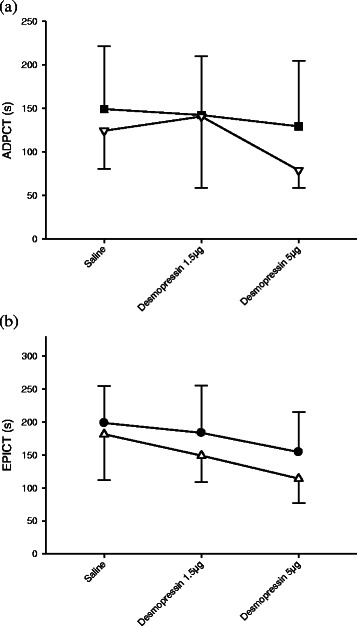
Effect of different doses of desmopressin on PFA-100® on ADP closure time (ADPCT (**a**)) at 32 °C (∎) and 37 °C (▽), as well as adrenaline closure time (EPICT (**b**)) at 32 °C (●) and 37 °C (△) respectively. * indicates *p* < 0.05. Error bars indicate SD

At 32 °C under the influence of aspirin, desmopressin produced a less pronounced effect on the closure times (Fig. 4). With desmopressin 1.5 microgram, both ADPCT and EPICT were not significantly different from their baseline values at 37 °C. At a dose of 5 microgram, desmopressin caused a mild but insignificant reduction of ADPCT when compared to baseline values at 37 °C (*p* = 0.431), while a significant reduction was only seen with EPICT (*p* = 0.011). The dose response curves of desmopressin did not seem to be temperature-dependent, as the slopes of dose–response curves are nearly parallel as shown in Fig. 5.

The vWF antigen did not show a significant increase after desmopressin 1.5 microgram and 5 microgram (Table 2). Other parameters did not show any significant change, except for haemoglobin level which showed a statistical significant (*p* < 0.05) but probably not clinically significant difference.

**Table 2:** Aspirin group - concentrations of von Willebrand factor antigen (vWF:Ag), haemoglobin (Hb), fibrinogen (FIB) and platelet count, in subjects receiving saline or desmopressin 1.5 and 5 μg subcutaneously

	Saline		Desmopressin 1.5		Desmopressin 5	
	(*n* = 10)		(*n* = 10)		(*n* = 10)	
	Baseline	Post	Baseline	Post	Baseline	Post
von Willebrand factor antigen; mU.ml^−1^	1051.3 ± 320.9	978.8 ± 509.5	918.6 ± 346.0	1028.8 ± 384.2	1025.6 ± 430.1	1266.5 ± 589.7
Hb; g.dl^−1^	14.4 ± 1.4	14.3 ± 1.3	13.9 ± 1.1	13.5 ± 1.0^+^	14.8 ± 1.4	13.9 ± 1.8^+^
Platelets; x 10^9^ l^−1^	218.0 ± 65.9	232.8 ± 52.6	222.0 ± 54.0	228.2 ± 32.3	258.9 ± 36.6	259.6 ± 41.2
FIB; mg.dl^−1^	332.0 ± 72.1	342.0 ± 69.6	337.1 ± 105.9	368.5 ± 75.1	364.4 ± 47.8	355.1 ± 53.4

Values in mean ± SD

^+^*p* < 0.05 within group post-treatment compared with baseline (Wilcoxon Signed-rank test)

While results from hypothermia group were presented in this paper, those from haemodilution group shall be presented in a separated report in the future.

## Discussion

The design of this study was based on our previous studies on evaluation of hypothermia-induced primary haemostasis impairment and the effect of desmopressin on such impairment [14, 17]. In this study, it was shown once again the baseline ADPCT and EPICT were significantly prolonged in presence of hypothermia. The addition of aspirin further prolonged the EPICT, showing a synergistic effect between hypothermia and aspirin on primary haemostasis impairment. As it is not uncommon to see patients taking aspirin peri-operatively, this may have clinical implication on peri-operative blood loss where hypothermia is difficult to avoid totally. In addition, it was previously shown that an EPICT >188 s could predict increased drain output in patients taking non-steroidal anti-inflammatory drugs after total knee arthroplasty with a sensitivity of 89 % [18]. EPICT prolongation in the presence of hypothermia and aspirin could therefore potentially predict an increase in peri-operative blood loss.

In our previous studies, we have shown that low dose subcutaneous desmopressin could improve hypothermia-induced impairment in primary haemostasis [17]. It was shown that the dosage required could be as low as 1.5 microgram, much lower than the standard dose of 15 microgram used in treating platelet dysfunction associated with uraemia or cirrhosis. In this follow-up study, it was shown that desmopressin at dosage of 1.5 microgram and 5 microgram could correct the ADPCT and EPICT to close to baseline values under the influence of hypothermia and aspirin. Such effect was milder at 32 °C when compared to 37 °C measurements. In fact, there seemed to be a trend for the 5 microgram dose to over-correct closure times to below baseline values (Figs. 2 and 3). Therefore we postulated that at dosage of 1.5 microgram desmopressin might be sufficient to cause improvement in hypothermia induced primary haemostasis impairment under the influence of aspirin.

Being a point-of-care monitoring of coagulation status, it has been suggested that the PFA-100® could be used as a means to monitor the effectiveness of aspirin therapy [19–22]. “Aspirin responders” and “aspirin non-responders” could be identified using population based cut-off values. In one particular study, Coakley et al. showed that PFA-100® could be used to identify “aspirin hyper-responders” peri-operatively [22]. This could allow us to identify a small group of “aspirin hyper-responders” who might be at risk of increased perioperative bleeding in presence of hypothermia and have their aspirin therapy evaluated before major operations. Indiscriminative cessation of aspirin therapy peri-operatively might unnecessarily pose the patients to increased risk of thrombotic events. PFA-100® could also be used as a screening tool for primary haemostasis abnormalities before surgery [23]. Although it is worth noting that studies so far had failed to show any clinical benefit of using PFA-100® (such as prediction of increased peri-operative blood loss) in peri-operative situations [24, 25].

The thrombotic risk of desmopressin could be related to its ability of causing prolonged von Willebrand factor (vWF) release. Our data showed that at the doses used in this study, there was no significant increase in vWF release. This is in concordance with our previous studies where low dose desmopressin corrects primary haemostasis impairment without causing an increase in vWF levels [17]. In an *in vitro* setting, the absence of endothelial cells means that there will not be vWF release accounting for the improvement in primary haemostasis. These suggested that desmopressin has direct effect on platelets itself, possibly via stimulated expression of adhesion molecules such as glycoprotein Ib and p-selectin [26–30]. Our studied dosages might allow safe and effective use of desmopressin on improving primary haemostasis impairment while minimizing the risk of thrombotic complications.

There are several limitations of our study. Firstly, this is an *in vitro* study based on healthy volunteers. Blood samples were first collected then incubated to 32 °C and 37 °C respectively. The *in vitro* setting may not reveal *in vivo* effects of desmopressin in hypothermic patients. Previous studies on the effectiveness of desmopressin on reduction of peri-operative blood loss had also shown mixed results [31–33]. In addition, whether desmopressin could improve haemostasis impairment due to other causes (e.g., haemodilution, acidosis, noval anti-platelet agents) remained elusive.

## Conclusion

In conclusion, aspirin could have a synergistic effect with hypothermia on primary haemostasis impairment. Such impairment could be reversed by subcutaneous low-dose desmopressin (1.5 microgram). This holds a promising and safe way to improve primary haemostasis impairment in aspirin taking patients undergoing major operations, where hypothermia may be difficult to avoid. Large scale clinical trials are warranted to further evaluate the efficacy and safety of desmopressin on this aspect. In addition, the PFA-100® might have a role in identifying “aspirin hyper-responders” who might be at risk of increased surgical bleeding. This could allow selective identification of high risk groups and have their peri-operative anti-thrombotic therapy reviewed, without risking the majority of thrombotic events with indiscriminative cessation of aspirin therapy.

## Abbreviations

ADPCT: Closure Time of Collagen/Adenosine Diphosphate
EPICT: Closure Time of Collagen/Epinephrine
PFA: Platelet Function Analyzer
vWF: von Willebrand Factor
RANOVA: Repeated measures analysis of variance
